# A Case of Penetrating Wound of the Chest; Pleuritis; Recovery

**Published:** 1853-05

**Authors:** James Knapp

**Affiliations:** Louisville, Ky.


### WESTERN JOURNAL

OF

### MEDICINE AND SURGERY.

MAY, 1853.

Article I.—*A Case of Penetrating Wound of the Chest; Pleuritis; Recovery.* By James Knapp, M. D., of Louisville, Ky.

Johnson Boyles, aged thirty-two years, of good constitution, was stabbed, December 18, 1852, at 8 o'clock, P. M., with a large knife, which penetrated the left side of the chest, severing the cartilage and adjoining intercostal spaces of the third rib, about one-third of an inch from the sternum. He supported himself for a moment against the wall, and, soon observing the sound produced by the passage of air through the wound, made pressure with the hand over the seat of injury, and asked to be helped home, a distance of two squares. By the support of a friend he was able to proceed one square, when he became faint. He was then placed in a recumbent posture and carried home.

I saw him about thirty minutes afterwards. There had been considerable external discharge from the wound; air had freely entered through the wound into the chest; left lung was collapsed; no expectoration of blood; pulse very weak; countenance pallid. I directed him to be placed upon the left side, so that the discharge might be external, and ordered an anodyne.

19th, 7, A. M.—Hemorrhage entirely ceased. Approximated the edges of the wound with stitches and strips of adhesive plaster, and directed the bowels to be moved.

12, M.—Reäction has taken place, and he complains of pain in the left side of the chest, more violent about the heart and the left shoulder; no respiratory murmur upon the injured side, but at each impulse of the heart a well-marked splashing sound could be heard in the præcordial region, that resembled nothing so much as the beating of the heart in a fluid — evidently blood. The patient was placed in a sitting posture, and four ounces of blood were abstracted from the arm, which produced a tendency to syncope, and afforded much relief.

4, P. M.—Prof. Gross attended with me in consultation. Pulse 90, regular, save an intermission about every tenth beat, sufficient volume, easily compressed; respirations twenty. Pericardium supposed to be wounded, and that the splashing sound, which could be heard at the distance of several feet from the bed, was produced by the blood within the pericardium. Prescribed sulphate of magnesia and senna, to be repeated until the bowels are more freely moved; also tartrate of antimony and potass, and sulphate of morphia, ä ä grs. $\frac{1}{8}$, to be administered every two hours; and directed a blistering plaster, six inches square to the anterior left side of the chest. Diet very light.

20th.—Prof. Gross in consultation. No improvement; splashing sound as distinct as yesterday. Bowels have been freely moved; blister has drawn well. Continue the tartarized antimony.

21st.—Prof. Gross with me. Resting more comfortably; voice improved; can take a fuller inspiration; no respiratory murmur upon the left side; fluid sound almost entirely gone. Directed the bowels to be kept freely open with sulphate of magnesia, and continued the tart. antimony.

January 1st.—Quite feverish; skin hot; countenance flushed; tongue more coated; pulse one hundred and thirty, and too weak to justify the use of the lancet; respirations twenty-three; tendency to cough. Prescribed hydrargyri chlor. mit. grs. viij., in divided doses, and continued the antimony.

6th.—Slept none last night; cannot remain recumbent on account of greater dyspnea, but lies upon his back, with the shoulders considerably elevated; great anxiety; says he has pain every where; blister still open. Removed the strips of adhesive plaster and examined the wound. Union not quite complete. The patient's position being changed so as to bring the wound more pendent, about three pints of sanguineous serum was discharged through the wound. Feels greatly relieved, and is able to resume the recumbent posture. The wound left open, with a few folds of linen placed over it, and the patient directed to so change his position several times each day, that the fluid might be discharged. Discontinued the antimony and directed an anodyne.

7th.—Rests comfortably; is more debilitated, and much emaciated. Directed porter, essence of beef, and toast.

9th.—Says he has no pains in the chest, but complains of much pain in the bowels, with dysenteric dejections. I considered that the intestinal irritation would prove beneficial by diverting morbid action from the thoracic organs, and as it was not sufficiently active to endanger the patient, contented myself with the administration of an occasional opiate, for the purpose of causing him to rest more comfortably. It is supposed that, since the 6th inst., two pints of serous fluid have been discharged through the wound.

12th.—A friend of the patient fearing that the dysenteric symptoms might endanger the patient, desired a consultation. Dr. Gross being absent from the city, Dr. T. S. Bell was called. Pulse ninety-eight, regular, of sufficient volume, easily compressed; respirations seventeen; wound discharging four ounces a day of sero-purulent matter; blister nearly healed. The irritation of the bowels considered favorable, and as the patient was able to sit in a chair two hours at a time, and was evidently doing well, no change was made in the treatment.

23d.—Sits up all day and walks about the room. Improving in flesh, and looks well. The discharge from the wound has continued to diminish until it has nearly ceased.

March 4th.—Has been on a visit to the country, seventy miles distant. Returned much improved in flesh and strength. Weighs one hundred and forty pounds; usual weight one hundred and fifty-five pounds. Has, at several different times, slowly walked ten squares. Says exercise soon produces palpitation and accelerated respiration; that the left shoulder and arm are weak, and that he frequently has pain in the shoulder, extending down the arm. Upon viewing the chest anteriorly upon the injured side, the wound is entirely healed; the pectoralis major muscle appears atrophied; the region of the third and fourth ribs considerably depressed; the respiratory movements less manifest than is natural, and the heart's action seems quite tumultuous; yet upon applying the ear to the præcordial region, the sounds of the heart are natural and the pulsations regular. The respiratory murmur is feeble over the entire left side of the chest; feebleness more marked anteriorly. Upon percussion there is a very perceptible dulness, more manifest upon the anterior than upon the posterior portion.

March, 1853.

